# Supplementary material for: Exosomally Targeting microRNA23a Ameliorates Microvascular Endothelial Barrier Dysfunction Following Rickettsial Infection
Source: Front Immunol. 2022 Jun 23;13:904679. doi: 10.3389/fimmu.2022.904679 (PMC9260018; doi:10.3389/fimmu.2022.904679)
Supplement: Supplementary file 1 [file DataSheet_1.pdf]

Supplemental Materials

Materials and Methods

Cell culture and *R. parkeri* infection

HUVECs (Cell Applications, Atlanta, GA) and human BMECs (iXCells, San Diego, CA) were cultured in Endothelial Cell Growth Medium (Cell Applications) at 37°C with 5% CO_2. Media were changed every two days. Cells were grown to 90% confluency prior to application of rickettsial inoculants. HUVECs were infected with *R. parkeri* at a MOI of 10. Uninfected ECs were used as mock-infected controls. BMECs were exposed to Exos at specified doses.

Assessment of delivery of miR23a into normal recipient BMECs

Following incubation with PKH26, the exosomes were washed three times with PBS by ultracentrifugation at 100,000 × *g* for 20 min at 4°C to remove the unbound stain. Moreover, we also introduced a Cy3-labeled mimic that was loaded into exosomes using $CaCl_2$ (final concentration 0.1 M) to trace the delivery of microRNA by exosomes. BMECs were exposed to the labeled exosomes for 12 hrs prior to fixation with 4% paraformaldehyde and staining with Hoechst or DAPI dye. Fluorescent images were visualized using an Olympus BX41 microscope.

Stem loop real time PCR

Total RNA was extracted from the same particle quantity of EVs using TRIzol® Reagent (Invitrogen, Thermo Fisher Scientific). An exogenous synthetic microRNA, namely cel-mir-39, was diluted in TRIzol before extraction to act as a normalizer. The concentration of total RNA was measured using a NanoDrop™ spectrophotometer (model ND-2000, Thermo Fisher Scientific). TaqMan™ MicroRNA Reverse Transcription kits (Thermo Fisher Scientific) were used for reverse transcription reactions. The 15 uL RT reactions contained 5 ng total RNA template, 3 uL RT Primer (5×), 0.15 uL dNTPs (100 mM), 1 uL MultiScribe™ reverse transcriptase (50 U/µL), 1.5 uL Reverse Transcription Buffer (10×), 0.19 uL RNase inhibitor (20 U/µL), and 4.16 uL nuclease-free water. Reverse transcription conditions were 16°C for 30 min, 42°C for 30 min, and 85°C for 5 min. For PCR amplification, the 10 uL PCR reactions included 0.7 uL cDNA template acquired above, 0.5 uL TaqMan™ Small RNA Assay Mix (20X), 5 uL PCR Master Mix, and 3.8 uL nuclease-free water. qPCR reaction conditions were 50°C for 2 min, 95°C for 30 sec, followed by 40 cycles of 95°C for 5 sec and 65°C for 30 sec. The relative expression of each miRNA was expressed as $2^{-(\triangle\triangle CT)}$ by the CFX 10 Connect Real-Time PCR Detection System (Bio-Rad, Hercules, CA).

Transendothelial electrical resistance (TEER)

TEER was measured using an electrical resistance meter (Millicell® ERS-2, Thermo Fisher Scientific), as reported[1]. BMEC monolayers were seeded on inserts in 24-well plates (0.4 µm polyester membrane, CoStar, Thermo Fisher Scientific), and TEERs were measured following various treatments as indicated. The values are shown as $\Omega \times cm^2$ and were normalized by subtracting the background (i.e., TEER measurement from an insert without cells).

Fluorescein isothiocyanate-dextran permeability assay[2]

Briefly, BMECs were plated in 6.5 mm diameter Transwell® polycarbonate membrane inserts (0.4 µm pore size, Costar, Corning) in 24-well plates. The confluent monolayers were treated as specified. Permeability was assessed following addition of fluorescein isothiocyanate-conjugated (FITC) dextran (FD40, MW 40,000, Sigma-Aldrich) to the apical compartment at 1 µg/ml. After 30 min the samples were collected from the basolateral compartment and measured on a fluorescence luminometer (Tecan, Morrisville, NC) at the wavelengths of 490 nm (excitation) and 520 nm (emission)[2].

Quantitative real time polymerase chain reaction (qRT-PCR)

Total RNA was extracted from BMECs by using TRIzol® Reagent (Invitrogen, Thermo Fisher Scientific). Purified RNA was then used for reverse transcription using EasyQuick RT MasterMix (CoWin Biosciences, Inc., Cambridge MA). The PCR reaction mixture volume was 20 μL, which included 10 μL of 2× Real Time PCR Master Mix (SYBR Green), 2 μL of cDNA template, 0.4 μL of forward primer (10 μM), 0.4 μL of reverse primer (10 μM), and 7.2 μL of sterile water. Relative expression of mRNA was expressed as $2^{-(\triangle\triangle CT)}$. GAPDH was utilized as a control to normalize gene expression data.

Immunofluorescence staining

BMECs were fixed with 4% methanol and incubated with anti-ZO-1 antibody (1:100, Zymed, Thermo Fisher Scientific) at 4°C overnight. After washing three times with phosphate-buffered saline/tween solution, all cells were incubated with Alexa Fluor 594-labeled goat anti-rabbit antibody for 2 h at room temperature. Nuclei were counterstained with DAPI. An IgG-matched isotype served as the internal control.

Western immunoblotting

For western immunoblotting, equal amounts of soluble protein were subjected to 10% SDS–polyacrylamide gel electrophoresis (SDS-PAGE). Proteins were transferred onto a polyvinylidene difluoride membrane and then incubated with primary antibody (1:1,000 for anti-Flotillin-1, albumin, AnnexinA2, CD63, calnexin-1[1], or ZO-1 antibodies) at 4°C overnight, followed by incubation with a secondary antibody at 1:10,000 for 2 hrs. Blots were visualized using the Pierce™ ECL Western Blotting Substrate kit (Thermo Fisher Scientific).

Supplemental Figure 1: A, Exo morphologies were verified using AFM height image (scale bars, 200 nm). B, Expression of protein markers in 100 μg protein derived from ECExos was examined using Western immunoblotting.

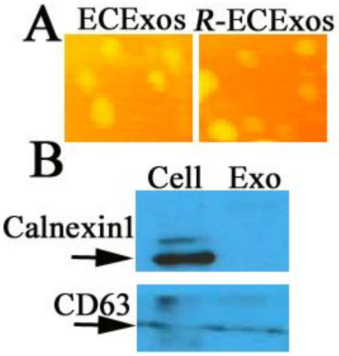

Supplemental Figure 2: Expression of total ZO-1 was examined using Western immunoblotting in BMECs.

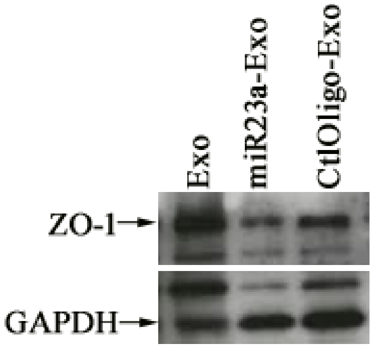

Supplemental Figure 3: Relative expression of ZO-1 mRNA was examined using RT-PCR in recipient BMECs. Compared to Ctl Oligo-enriched ECExos (CtlOligo-Exos), miR23a ASO-enriched ECExos (ASO-Exo) (1,000 particles/cell) attenuate *R*-ECExo-induced downregulation of ZO-1 mRNA in recipient BMECs. **, $p<0.01$.

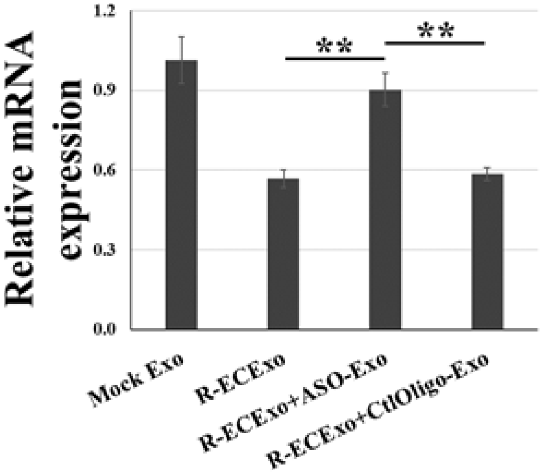

Supplemental Figure 4: A demonstration of the measurement of the endothelial LBF using fluidic AFM.

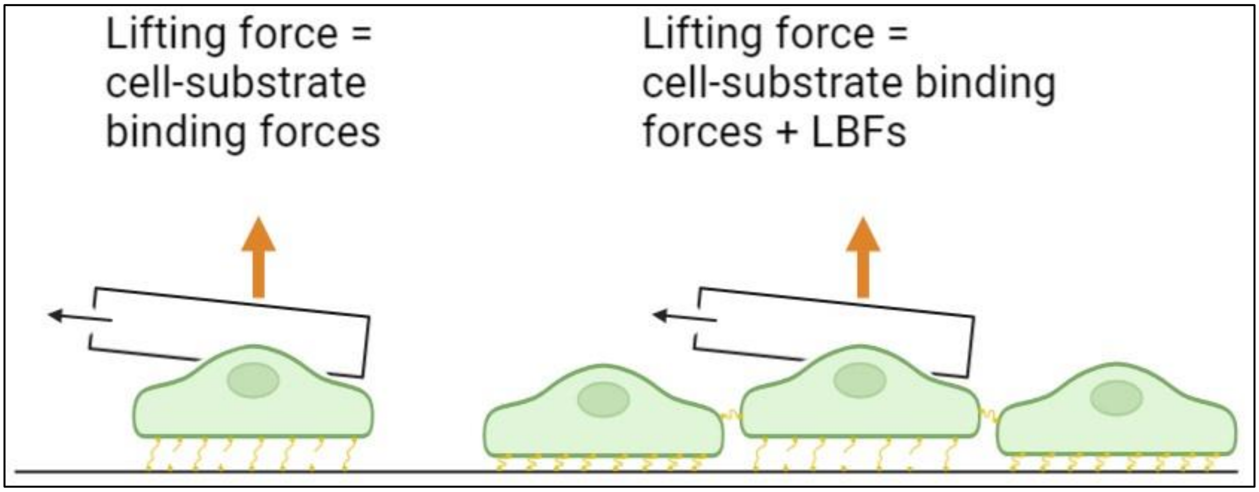

1. Liu Y, Zhou C, Su Z, et al. Endothelial Exosome Plays a Functional Role during Rickettsial Infection. *mBio*. May 2021;12(3)doi:10.1128/mBio.00769-21

2. Gong B, Lee YS, Lee I, et al. Compartmentalized, functional role of angiogenin during spotted fever group rickettsia-induced endothelial barrier dysfunction: evidence of possible mediation by host tRNA-derived small noncoding RNAs. *BMC Infect Dis*. 2013;13:285. doi:10.1186/1471-2334-13-285
